# Velocity-Based Resistance Training on 1-RM, Jump and Sprint Performance: A Systematic Review of Clinical Trials

**DOI:** 10.3390/sports10010008

**Published:** 2022-01-04

**Authors:** Mateo Baena-Marín, Andrés Rojas-Jaramillo, Jhonatan González-Santamaría, David Rodríguez-Rosell, Jorge L. Petro, Richard B. Kreider, Diego A. Bonilla

**Affiliations:** 1 Grupo de Investigación ZIPATEFI, Fundación Universitaria del Área Andina, Pereira 110231, Colombia; mbaena@utp.edu.co (M.B.-M.); jgonzalez@utp.edu.co (J.G.-S.); 2 Grupo Investigación y Desarrollo en Cultura de la Salud, Universidad Tecnológica de Pereira, Pereira 660001, Colombia; 3 Grupo de Investigación de Ciencias Aplicadas a la Actividad Física y el Deporte, Universidad de Antioquia, Medellín 050010, Colombia; andres.rojasj@udea.edu.co; 4 Physical Performance & Sports Research Center, Universidad Pablo de Olavide, 41013 Seville, Spain; davidrodriguezrosell@gmail.com; 5 Department of Sport and Informatics, Universidad Pablo de Olavide, 41013 Seville, Spain; 6 Research, Development and Innovation (R&D+i) Area, Investigation in Medicine and Sport Department, Sevilla Football Club, 41005 Sevilla, Spain; 7 Research Division, Dynamical Business & Science Society—DBSS International SAS, Bogotá 110311, Colombia; jlpetro@dbss.pro; 8 Research Group in Physical Activity, Sports and Health Sciences—GICAFS, Universidad de Córdoba, Montería 230002, Colombia; 9 Exercise & Sport Nutrition Laboratory, Human Clinical Research Facility, Texas A&M University, College Station, TX 77843, USA; rbkreider@tamu.edu; 10 Sport Genomics Research Group, Department of Genetics, Physical Anthropology and Animal Physiology, Faculty of Science and Technology, University of the Basque Country (UPV/EHU), 48940 Leioa, Spain

**Keywords:** resistance training, muscle strength, athletes, strength training, athletic performance, weight bearing strengthening program

## Abstract

Weight resistance training (RT) has been shown to positively influence physical performance. Within the last two decades, a methodology based on monitoring RT through movement velocity (also called velocity-based resistance training, VBRT) has emerged. The aim of this PRISMA-based systematic review was to evaluate the effect of VBRT programs on variables related to muscle strength (one-repetition maximum, 1-RM), and high-speed actions (vertical jump, and sprint performance) in trained subjects. The search for published articles was performed in PubMed/MEDLINE, SPORT Discus/EBSCO, OVID, Web of Science, Scopus, and EMBASE databases using Boolean algorithms independently. A total of 22 studies met the inclusion criteria of this systematic review (a low-to-moderate overall risk of bias of the analyzed studies was detected). VBRT is an effective method to improve 1-RM, vertical jump and sprint. According to the results of the analyzed studies, it is not necessary to reach high muscle failure in order to achieve the best training results. These findings reinforce the fact that it is possible to optimize exercise adaptations with less fatigue. Future studies should corroborate these findings in female population.

## 1. Introduction

Weight resistance training (RT) has been proven as an important tool to improve performance in different sports disciplines [1,2,3,4]. In addition, RT is also a key factor to improve body composition in physically active population [5,6], health condition in the general population [7,8], and even as preventive and palliative treatment in many metabolic and neurodegenerative diseases [9,10]. The magnitude of the effect produced by RT will depend on the manipulation of the so-called acute training variables, especially the relative load, volume, and type of exercises [11,12,13]. Traditionally, relative load during RT has been configured using the percentage of one-repetition maximum (1-RM) or the maximum number of repetitions complete against an absolute load (X-RM) [11,12]. Both methodologies present several limitations related to excessive degree of fatigue, spending time, and difficulty in applying tests of these characteristics to certain populations, such as the elderly, young people, and athletes without previous experience in RT [14,15]. However, the main limitation of the 1-RM percentage-based RT (PBT) method to prescribe the relative load is the mismatch between the actual load and the proposed load that occurs as a result of daily changes in 1-RM [16]. As a consequence, it is not possible to know the real load with which the athlete has trained in each training session and, therefore, it is impossible to determine the load that has produced a certain training effect. For example, a pre-specified 1-RM value during a long-term training program disregards a number of confounding factors (i.e., sleep, diet, and training-induced fatigue) that affect the athlete’s “true” load and daily preparedness [17]. On the other hand, volume is prescribed in terms of sets and repetitions per set [12]. However, the maximal number of repetitions completed against a given relative load present high between-subjects variability [18]; thus, subjects performing the same number of repetitions per set against the same relative load are likely performing a different effort [18,19].

To solve these problems of monitoring and quantification of the training loads, recent attention has been placed on movement velocity during RT [14,20,21]. First, several studies have observed a strong relationship between the relative load (1% RM) and movement velocity in different resistance exercises [22,23,24]. Thus, it is possible to estimate the 1% RM that is being used as soon as the first repetition with any given absolute load is performed [14]. Furthermore, there is also a strong relationship between the percentage of velocity loss (VL) attained in the set and the percentage of repetitions completed with respect to the maximum possible repetitions against a given relative load [18,19]. These results suggest that, rather than prescribing a fixed number of repetitions with a given relative load, training volume during RT should be monitored using the magnitude of VL attained in each exercise set because it is closely linked to the actual level of effort being incurred [18,19,25,26]. In addition, it has been observed that, for the same relative load, the magnitude of VL incurred in the set determine the degree of fatigue [27,28]. Therefore, first repetition’s mean velocity (which is intrinsically related to the 1% RM being used) and the percent VL to be reached during each set are the two variables that might be prescribed and monitored during an RT program aimed to optimize athletic performance. This procedure for monitoring and dosing the load is called “velocity-based RT” (VBRT) [27,29,30].

Previous studies, systematic review, and meta-analysis have shown greater strength gains for RT using repetitions not to failure compared to muscle failure [31,32,33]. Additionally,, this type of RT induced lower metabolic stress and endocrine response [27,34]. Despite these findings, the knowledge concerning the influence of different level of efforts on strength capacity and other variables related to athletic performance is limited. In an attempt to answer this fundamental question, some studies have shown the importance of VBRT method for monitoring and adjusting the fatigue degree in real time in order to find the optimal training load for improving the physical performance [28,35,36]. In this regard, it has been reported that VBRT generates significant improvements in the rate of force development (RFD) of trained athletes, which would directly influence athletic actions, such as jumps and sprints [37]. However, the magnitude of training-effects is determined by the degree of fatigue induced during RT program [29,30,38,39,40]. If the athlete presents higher movement velocities against the same absolute load, this indicates an improvement of the maximum strength and the RFD against that load. In fact, a recent meta-analysis with meta-regression highlighted this and concluded that the application of faster movement velocity and the intention to produce rapid force result in the greatest increases in RFD [41]. Similarly, the VBRT allows the evaluation and control of the magnitude of VL during a set, which is an indicator of increased muscle fatigue. This fact is the special interest for the specificity of the stimulus since the fact of performing slower repetitions would not only indicate fatigue (as a consequence of reaching a higher percentage of VL in the series), but would also be could also lead to suboptimal adaptations [22,24,42].

Although the application of the VBRT has been shown to improve training in high-performance athletes [43,44] some studies have found no superior effects when individualizing based on the force-velocity profile [45]. In addition, with the emergence of new brands of linear position transducers, accelerometers, and applications for mobile devices [46,47], VBRT has become more accessible to the general public and has gone from being applied in the laboratory to become a tool for the personal trainer who seeks to control and program the training load quickly and safely. Considering that an analysis of the scientific literature regarding the effect of VBRT in elite or recreationally trained populations aiming to improve muscular strength and physical performance has not been carried out, the goal of this systematic review is to evaluate the effect of adjusting and controlling loads within a VBRT program on physical performance variables related to muscular strength and high-speed actions (maximum repetition [1-RM], vertical jump, and running speed) in trained subjects.

## 2. Materials and Methods

### 2.1. Protocol and Registration

This systematic review was developed and reported according to the parameters established in the Preferred Reporting Items for Systematic reviews and Meta-Analyses (PRISMA) [48]. Considering that this review is not eligible to be registered in PROSPERO, as it focused on physical performance, the protocol of this work has been published in Figshare to make it publicly accessible and avoid unnecessary duplication of analyzing the same study objective (https://doi.org/10.6084/m9.figshare.13065950).

### 2.2. Eligibility Criteria

The studies were selected according to the following inclusion criteria: (1) randomized clinical trials; (2) published in specialized scientific journals hosted in databases from 2009 onwards; (3) written in English or Spanish; (4) with full-text access; (5) that evaluated VBRT using either an alternative experimental group or a control group (traditional 1-RM percentage-based training); and (6) that reported effects on variables related to muscle strength (1-RM), vertical jump, or running speed. We excluded articles that were not original studies (i.e., editorials, notes, perspectives, narrative reviews, dissertations, etc.), interventions of less than four weeks, and studies including subjects with RT experience less than six months.

### 2.3. Information Sources

The search for published articles was carried out in the databases PubMed/MEDLINE, SPORTDiscus/EBSCO, OVID, Web of Science, Scopus and EMBASE.

### 2.4. Search Methods

The PICO strategy was used: P (*athletes*) I (*VBRT*) C (*resistance training*) O (*strength, jump and sprint performance*), which allows the problem to be analyzed according to Patient, Intervention, Comparison, and Outcomes [49] to structure the search strategy. The authors (M.B.-M. and A.R.J.) independently ran the following Boolean algorithms to search for studies in each database: PubMed/MEDLINE, SPORTDiscus/EBSCO, OVID y Web of Science: velocity based training AND athlet* AND “resistance training” AND strength NOT old NOT elder*; Scopus: *“velocity based” training AND “resistance training” AND strength*; and EMBASE: velocity AND based AND training AND athlet* AND ‘resistance training’ AND strength NOT old* NOT elder*. Additionally, free language terms related to “*velocity-based resistance training*” were used to complement the search in some databases.

### 2.5. Study Selection

After performing the search, the filter option of the databases was used to meet the inclusion criteria 1 to 4. Subsequently, the remaining publications were manually filtered in an Excel matrix where the title of the article, year of publication, name of the journal, abstract, objective, conclusions were structured. The study selection process took place during February and May 2021 although an updated search was conducted prior to manuscript submission (October 2021).

### 2.6. Data Collection Process and Items

The authors (M.B.-M., A.R.J., and D.A.B.) were responsible for extracting the following information for subsequent analysis: (i) reference and year of publication; (ii) descriptive statistics of the study population (number of subjects, sex, age, and BMI); (iii) description of the experimental intervention; (iv) assessment methods, magnitude and units of the variables related to muscle strength and power; and (v) baseline values and change in the study variables.

### 2.7. Risk of Bias

The risk of bias of the selected publications was assessed with the Cochrane risk of bias analysis tool RoB 2.0 [50] including: selection bias, performance bias, detection bias, attrition bias, outcome reporting bias, and any other bias. Discrepancies were identified and resolved through discussion among the authors (with the intervention of a third author when necessary). All randomized participants were included in the analysis of each study, considering that this is the least biased way to analyze clinical effects. Figures to represent the results of the risk of bias assessment were developed using the package Risk-of-bias Visualization (robvis) [51].

### 2.8. Data Analysis and Synthesis

Changes in variables related to muscle strength (1-RM), vertical jump or running speed after intervention with a VBRT program were considered as the primary outcome. Those selected publications that met all the inclusion criteria went on to the next phase of analysis and synthesis, where a table was used to report the comparison of results.

## 3. Results

### 3.1. Study Selection

**Figure 1:**
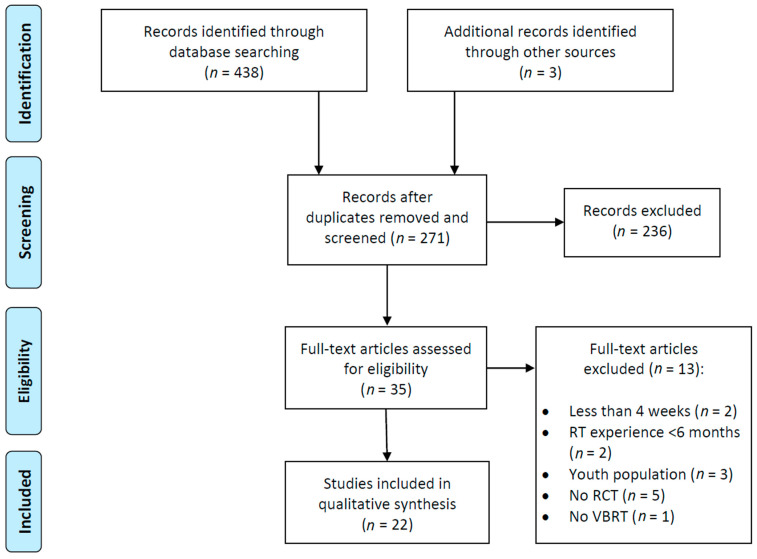
PRISMA flow diagram.

After running the search algorithms with Boolean operators and free language terms, 441 references were retrieved. Then, the screening process of the publications was performed (filtering by date, type of article and availability of full text) and 271 potentially eligible studies were found. However, after evaluating the abstracts, full-texts, and analyzing the strict fulfillment of the other inclusion criteria, 236 articles were excluded. A total of 22 studies met the pre-established requirements [22,29,30,38,39,40,52,53,54,55,56,57,58,59,60,61,62,63,64,65,66,67]. Considering that the present systematic review is reported according to the parameters established in the PRISMA guidelines, a flow chart of the literature search is shown in Figure 1.

### 3.2. Risk of Bias within Studies

**Figure 2:**
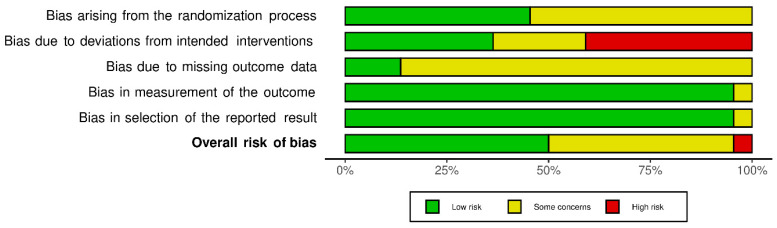
Risk of bias summary for included studies. Weighted bar-chart of the distribution of risk-of-bias judgments. These graphics were obtained using the ‘robvis’ package within the R statistical computing environment.

The methodological quality of the selected studies is presented in Figure 2.

### 3.3. Results of Individual Studies

The main characteristics and results of the selected studies are shown in Table 1.

## 4. Discussion

### 4.1. Effects on 1-RM

A high percentage of the analyzed articles (95.45%) have evaluated the effects of VBRT on 1-RM (Table 1). Changes in this variable have been reported in several exercises, such as the squat, bench press, pull-ups, overhead press, and deadlift with full or partial ranges of motion. Most of the studies evaluating the squat exercise reported similar or higher significant improvements in the 1-RM for the VBRT group in comparison to control groups. In fact, only one study did not find positive effects after a VBRT protocol with 30% of VL [38]. Moreover, the research by Jiménez-Reyes et al. [67] showed that non-adjusted loads during a VBRT program resulted in greater changes in 1-RM than adjusting training loads although the improvements were probably due to the former group working at lower relative intensity (with lower VL). According to the authors, this might have generated lower fatigue levels and greater performance benefits. By comparing the effects of PBT and VBRT programs, Banyard et al. [60] found that both training methods might increase 1-RM although higher clinical significance (larger effect size) favored PBT in stronger individuals while VBRT was more advantageous for improving high-speed actions (e.g., CMJ, sprint). Notwithstanding, most of the studies revealed greater improvements in 1-RM after a VBRT program when compared to PBT (Table 1). Regarding to exercise technique, Pallares et al. [63] evaluated three different positions of the squat exercise: full squat, parallel squat, and half squat. This study showed statistically significant effects in full and parallel squat, but not in half squat. These findings are consistent with the fact that most of the studies reviewed have found positive results in full squat after a VBRT program [22,29,30,38,39,40,53,55,60,62,65,66,67].

Eight studies have used the bench press as an intervention exercise for the evaluation of the VBRT effects on strength-related variables. Interestingly, it has been reported that performing this exercise in a partial range of moment does not generate positive effects during VBRT-based programming in recreational and well-trained athletes [58]. In fact, it is worth noting that this research evaluated different ranges of movement during bench press (full, two-thirds, and one-third bench press) without finding significant effects in the group that trained at one-third of the movement and minor effects in those subjects who performed partial bench press [31].

In addition, the programmed pull-up exercise through the movement velocity was evaluated in a study conducted by Sánchez-Moreno et al. [61]. This study showed that the group with 25% of VL obtained significant improvements in 1-RM while no changes were observed during RT close to muscle failure (50% VL) [54]. On the other hand, although VBRT-based programming in the overhead press has also shown significant improvements in 1-RM values, no improvements have been described for the deadlift [62]. In general terms, VBRT has been shown to be efficient in improving strength measured through 1-RM in most exercises. However, when RT involved exercise executed in a partial range of motion, the training effect is considerably less.

### 4.2. Effects on Sprint Performance

Fifteen studies have investigated the effect of VBRT on running sprinting (10–30 m) with a total of 406 strength-trained subjects [22,29,30,38,39,40,52,55,57,59,60,63,64,66,67]. However, only eight studies [29,30,39,55,59,60,66,67] concluded that VBRT has positive or superior effects to other strategies, while the other studies did not report improvements with respect to the control group. Despite these findings, it should be noted that VBRT does not negatively affect the sprinting ability of athletes. As expected, one of the most interesting findings of most studies was that positive results are found in the groups training at a higher movement velocity during the concentric phase (i.e., lower relative loads). In addition, there was a tendency to show greater increases in sprint performance when less percentage of VL was used in each training set. This resulted in lower total volume than the other groups and, therefore, a lower level of accumulated fatigue [29,30,39,55,59,60,66,67]. Although some studies have reported no significant difference in sprinting ability when comparing two groups training with different VL [22,38], several studies suggest that lower %VL (10–15%) could be the most appropriate fatigue level to improve 20 m sprint performance [29,30,39]. Moreover, compared to PBT, the VBRT methodology showed more benefits on the performance of sport-specific actions, such as sprints and changes of direction [60]. Finally, it seems that the full squat [63] with feedback [52] would be most recommended. In any case, it seems that more research is needed regarding the effect of different ranges of motion in lower-limb exercises during a VBRT program that seeks to improve sprint performance.

### 4.3. Effects on CMJ

Of the selected studies, only three did not evaluate the effect of a VBRT program on countermovement jump (CMJ) [54,58,61]. Thus, results are analyzed for a total of 363 subjects with experience in RT. The findings indicate that VBRT positively affects the CMJ of athletes, obtaining benefits in functional performance with lower total training load. Similar to sprint performance, it appears that the most efficient VBRT program would be the one with lower percentage of VL in the set [22,38,39,40,57] and performed at maximum intended velocity [55], as it would reduce fatigue accumulation and facilitate motor neuronal adaptations to improve the ability to apply force quickly. Similar to the other strength-related variables, the full range of motion [63] with feedback [52,64] seems to show the best results on CMJ.

### 4.4. Velocity Loss

Several studies have implemented the VL in the set (from 5% to 45%) to program the training volume in different exercises, the squat being the most used [22,29,30,38,39,40,62,65,66]. In general, it is concluded that it is not necessary to reach a high percentage of VL in the squat exercise, as magnitudes of VL ranging from 5 to 20% appear to show the best results on jump, sprint, and strength performance (see Table 1). Therefore, results of these studies suggest that exercise adaptations are favored with a lower total training volume and less fatigue for the athlete. Some studies have evaluated biochemical markers of muscle damage and electromyography activity during a VBRT program with different VL in the set [29,30,65]. Although the concentrations of certain biomarkers did not show important differences, a significant increase in troponin T (a marker of muscle damage) was observed for training groups with 30% of VL compared to a 10% of VL in the set [30]. These results are in agreement with those reported by Pareja-Blanco et al. [38,68] who indicated that the degree of fatigue attained at VL below 10% could be an important factor in optimizing neuromuscular adaptations during a VBRT program. Regarding the muscle activation assessment, no significant differences were found between the pre and post-values; however, a VL in the set equal to 10% presented better percentage changes with respect to the 30% group in muscle fiber recruitment, mean, and peak power frequency [30]. In summary, the magnitude of VL in the set is a variable that takes center stage in VBRT. Studies have shown that it is not necessary to generate high magnitude of VL in the set (>20%) to increase muscle strength in exercises such as squat [57], pull-ups [61], and bench or overhead press [62]. Thus, the results of several studies suggest that higher training volumes do not always bring better results when seeking to increase strength levels, which gives some relevance to lower accumulated fatigue during neuromuscular adaptations.

### 4.5. Limitations and Future Directions

Despite the fact that sports performance variables were measured, the samples of the selected studies did not always include performance athletes (particularly, some studies included physically active young adults between 20 and 30 years old). Additionally, we are aware that the analyzed studies did not have a “true” control group (that is a group that did not receive treatment and therefore did not train); notwithstanding, we have based our analysis on pre-post changes and compared this (when available) to either alternative experimental groups or traditional 1-RM percentage-based training groups (control group). Although different authors have reported that improvements in strength-related variables are associated with sports performance, such as vertical jump, running speed, and throwing speed [69,70,71,72], it is worthwhile to evaluate the effect of this methodology on specific technical gestures of different sports modalities [73]. So far, it can only be stated that VBRT has positive effects on the performance of exercises such as the squat, bench press, pull-ups, and overhead press. Furthermore, the study designs showed some heterogeneity, low-to-moderate risk of bias and have mainly included male participants (training experience from 1.5 to 8.5 years), which suggests that more research is needed to evaluate the effects of this methodology in the female population. On the other hand, it has been shown that not all exercises obtain the best performance benefits from the same velocity losses, which implies that movement velocity is exercise-dependent [74]. Thus, future studies should analyze the effect of VBRT in a larger number of exercises, with free weights or other types of resistances such as elastic bands, and explore the potential effects of different RT programming approaches on strength-related variables. Indeed, only two studies have evaluated different methodologies (e.g., linear, undulating, or reverse) during a VBRT program with no relevant differences between them [65,66]. Finally, although new devices and smartphone applications have been developed recently [46,47], further external validation research is required.

### 4.6. Practical Applications

Evidence support the fact that PBT improves maximal strength. However, additional training methods such as VBRT might optimize load prescription and exercise adaptations [75]. Considering the main findings of the selected studies, VBRT has proven to be an effective methodology for improving strength in RT-trained individuals while completing lower training volumes compared to traditional programming. The following are some practical recommendations based on previous articles [21,76] and our systematic review:

- A VBRT program with three sets per exercise performed twice a week might have significant changes in the 1-RM, CMJ, and sprint performance for a period of eight weeks;
- Training at maximum intended concentric velocity is a key requirement to optimize muscle strength and gains in high-speed actions using the VBRT methodology;
- It is necessary that the complete rest (>3 min) between sets are necessary to work at the maximum intended velocity at high intensity;
- The load-velocity profile and mean propulsive velocity are valid options for monitoring intensity and load progression;
- The magnitude of VL in the sets is a practical and objective method to program the training volume during RT;
- A high magnitude of VL in the set is not necessary to achieve the best results on muscle strength and athletic performance. In fact, lower velocity losses (5–10%) guarantee less fatigue accumulation, which might lead to quicker recovery.

## 5. Conclusions

Movement velocity is a useful and reliable variable for monitoring and adjusting the training loads during RT, making it easier for the personal trainer to program workloads consistent with the athlete’s current performance capacity. This procedure corrects one of the main shortcomings of PBT, where the programming of the loads is based on the 1-RM of the athlete, ignoring the fact that this measure presents a high variability throughout the training sessions. After reviewing current scientific evidence, VBRT seems to be an alternative and efficient methodology to improve muscle strength (1-RM), CMJ and sprint performance, although more research is needed in female participants to corroborate these results. It must be noted that to improve muscle strength and high-speed actions, the RT should not induce a high VL during the set or reach muscle failure. These results reveal that it is possible to obtain more gains with less fatigue.

## Figures and Tables

**Table 1:** Evidence of the effects of a velocity-based resistance training program on muscle strength/power.

Reference	*n* (M:F)	Age, Body Mass and Stature	VBRT Program						Analyzed Variable and Change (%)	
			Groups (*n*)	Frequency (Days·wk^−1^)	Intensity	Volume	Duration (Weeks)	Device		
Randell et al. [52]	13 (13:0)	25.7 ± 3.6 years; 188.5 ± 8.2 cm; 104.3 ± 10.0 kg	G_VBRT1_ (7): feedback G_VBRT2_ (6): no feedback	3	40 kg 4–6 RM	3–6 reps × 3–5 sets	6	Celesco PT5A-150	CMJ (cm)	G_VBRT1_: 4.6 G_VBRT2_: 2.8
									Horizontal Jump (cm)	G_VBRT1_: 2.6 * G_VBRT2_: 0.5
									Sprint 10 m	G_VBRT1_: 1.3 G_VBRT2_: 0.1
									Sprint 20 m	G_VBRT1_: 0.9 G_VBRT2_: 0.1
									Sprint 30 m	G_VBRT1_: 1.4 * G_VBRT2_: −0.3
Ramos et al. [53]	27 (27:0)	20.4 ± 5.0 years; 180.3 ± 5.9 cm; 81.4 ± 8.4 kg	G_VBRT_ (16) G_control_ (11)	2	MPV: 0.7–1	4–8 reps × 3–4 sets	18	T-Force	CMJ (cm)	G_VBRT_: 6.9 ^†^ G_control_: 2.5
									RM_BP_ (kg)	G_VBRT_: 10.5 ^†^ * G_control_: 4.9
									RM_SQ_ (kg)	G_VBRT_: 14.2 ^†^ * G_control_: 3.4
Gonzalez-Badillo et al. [54]	20 (20:0)	21.9 ± 2.9 years; 177 ± 8 cm; 70.9 ± 8.0 kg	G_VBRT_ HV (9) G_control_ MV (11)	3	MPV: 0.79 (HV) 0.47 (MV)	2–6 reps × 3–4 sets	6	T-Force	1-RM (kg)	G_VBRT_: 18.2 * G_control_: 9.7
Pareja-Blanco et al. [55]	21 (21:0)	23.3 ± 3.2 years; 177 ± 7.0 cm; 73.6 ± 9.2 kg	G_VBRT_ HV (10) G_control_ MV (11)	3	MPV: 1.0 (HV) 0.84 (MV)	2–8 reps × 3–4 sets	6	T-Force	CMJ (cm)	G_VBRT_: 8.9 * G_control_: 2.4
									Sprint 10 m	G_VBRT_: −2.8 * G_control_: −1.1
									Sprint 20 m	G_VBRT_: −1.6 G_control_: −1.6
									Sprint 30 m	G_VBRT_: 18 * G_control_: 9.7
Dolezal et al. [56]	19 (10:9)	19.9 ± 1.5 years; 178.9 ± 7.0 cm; 88.4 ± 19.5 kg	G_VBRT_ M (10) G_VBRT_ F (9)	3	50–80% 1-RM	2–8 reps × 3–6 sets	12	NA	RM_SQ_	G_VBRT_ M: 14.3 ^†^ G_VBRT_ F: 18.4 ^†^
Pareja-Blanco et al. [38]	22 (24:0)	22.7 ± 1.9 years; 176 ± 6 cm; 5.8 ± 7.0 kg	G_VBRT1_: 20% VL (12) G_VBRT2_: 40% VL (10)	2	MPV 0.85–0.62	3 sets with 20% or 40% VL	8	T-Force	RM	G_VBRT1_: 17.6 ^†^ * G_VBRT2_: 13.5 ^†^ *
									CMJ	G_VBRT1_: 9.1 ^†^ * G_VBRT2_: 3.7
									Sprint 20 m	G_VBRT1_: −0.3 G_VBRT2_: 1
Pareja-Blanco et al. [22]	16 (16:0)	23.8 ± 3.4 years, 174 ± 7 cm; 75.5 ± 8.6 kg	G_VBRT1_: 15% VL (8) G_VBRT2_: 30% VL (8)	3	MPV 1.13–0.82	3 sets with 15% or 30% VL	6	T-Force	RM	G_VBRT1_: 8.9 ^†^ G_VBRT2_: 3.6
									CMJ	G_VBRT1_: 5.3 ^†^ * G_VBRT2_: −2.6
									Sprint 30 m	G_VBRT1_: −0.5 G_VBRT2_: −0.2
Perez-Castilla et al. [57]	20 (20:0)	22.0 ± 0.2 years; 175.7 ± 1.5 cm; 77 ± 18.4 kg	G_VBRT1_: 10% VL (10) G_VBRT2_: 20% VL (10)	2	MPV 1.20	36 reps with 10% or 20% VL	4	T-Force	RM	G_VBRT1_: 2.0 G_VBRT2_: 3.6
									CMJ	G_VBRT1_: 6.3 ^†^ * G_VBRT2_: 3.6 ^†^
									Sprint 15 m	G_VBRT1_: 0.41 G_VBRT2_: 0.41
Martinez-Cava et al. [58]	50 (50:0)	24.0 ± 4.7 years; 176.2 ± 8.4 cm; 73.4 ± 9.9 kg	G_BP_ (11) G_BP2/3_ (13) G_BP1/3_ (12) G_control_ (12)	2	MPV 60–80% 1-RM	4–8 reps × 4–5 sets	10	T-Force	RM_BP_	G_BP_: 12.3 ^†^ G_BP2/3_: 7.01 G_BP1/3_: −0.26 G_control_: −2.9
Galiano et al. [39]	28 (28:0)	23.0 ± 3.2 years; 175.8 ± 4.7 cm; 73.8 ±10.8 kg	G_VBRT1_: 5% VL (15) G_VBRT2_: 20% VL (13)	2	MPV 1.14	3 sets with 5% or 20% VL	7	T-Force	RM	G_VBRT1_: 10.7 ^†^ G_VBRT2_: 13.6 ^†^
									CMJ	G_VBRT1_: 9.3 ^†^ G_VBRT2_: 8.8 ^†^
									Sprint 20 m	G_VBRT1_: −4.9 ^†^ G_VBRT2_: −3.6 ^†^
Orange et al. [59]	27 (27:0)	17 ± 1 years; 179 ± 5.8 cm; 83.15 ± 11.9 kg	G_VBRT_ (12) G_PBT_ (15)	2	60–80% 1-RM	5 reps × 5 sets	7	OptoJump Witty Timing System	RM	G_VBRT_: 0.38 ^†^ * G_PBT_: 0.51 ^†^
									CMJ	G_VBRT_: 0.53 ^†^ G_PBT_: 0.40 ^†^
									Sprint 5 m	G_VBRT_: −0.09 G_PBT_: −0.69 ^†^
									Sprint 10 m	G_VBRT_: −0.41 ^†^ G_PBT_: −0.81 ^†^
									Sprint 20 m	G_VBRT_: −0.48 ^†^ G_PBT_: −1.02 ^†^
									Sprint 30 m	G_VBRT_: −0.70 ^†^ G_PBT_: −0.78 ^†^
Banyard et al. [60]	24 (24:0)	25.5± 6 years 84.7± 6.8 kg	G_VBRT_ (12) G_PBT_ (12)	3	MPV 0.84–0.62	5 reps × 5 sets	6	NA	RM CMJ Sprint 5 m Sprint 10 m Sprint 20 m	G_VBRT_: 11.3 ^†^ G_VBRT:_ 7.4 ^†^ * G_VBRT_: −6.5 * G_VBRT_: −3.8 ^†^ * G_VBRT_: −1.8
Sanchez-Moreno et al. [61]	29 (29:0)	26.5 ± 6.3 years; 176.1 ± 5.3 cm; 74.3 ± 6.1 kg	G_VBRT1_: 25% VL (15) G_VBRT2_: 50% VL (14)	2	MPV 0.84	2–4 sets with 25% or 50% VL	8	T-Force	RM	G_VBRT1_: 5.4 ^†^ * G_VBRT2_: 0.7
Dorrell et al. [62]	16 (16:0)	22.8 ± 4.5 years; 180.2 ± 6.4 cm; 89.3 ± 13.3 kg	G_VBRT1_ (8) G_PBT_ (8)	2	MPV 0.51–0.91	3 sets with 20% VL	6	GymAware PowerTool	RM_SQ_	G_VBRT_: 9.3 ^†^ G_PBT_: 9.0 ^†^
									RM_BP_	G_VBRT_: 7.3 ^†^ * G_PBT_: 4.8 ^†^
									RM_OP_	G_VBRT_: 6.5 ^†^ G_PBT_: 6.1 ^†^
									RM_DL_	G_VBRT_: 6.3 ^†^ G_PBT_: 2.9
									CMJ	G_VBRT_: 4.9 ^†^ * G_PBT_: 1.0
Pallares et al. [63]	53 (53:0)	23.0 ± 4.4 years; 174.0 ± 7.4 cm; 76.0 ± 12.8 kg	G_VBRT1_ FSQ (12) G_VBRT2_ PSQ (13) G_PBT_ HSQ (11) G_control_ (14)	2–4	MPV 0.43–0.79	4–8 reps × 4–5 sets	10	T-Force	RM_SQ_	G_VBRT1_: 16.4 ^†^ G_VBRT2_: 10.3 ^†^ G_VBRT3_: 2.6 ^†^ G_control_: −8.08 ^†^
									PP	G_VBRT1_: 6.5 G_VBRT2_: 5.9 G_VBRT3_: −1.2 G_control_: −0.2
									CMJ	GVBRT1: 12.8 ^†^ GVBRT2: 9.1 GVBRT3: 5.3 G_control_: −3.4
									Sprint 20 m	GVBRT1: −2.4 GVBRT2: −1.0 GVBRT3: 0.0 G_control_: 1.7
Pareja-Blanco et al. [40]	55 (55:0)	24.1 ± 4.3 years, 175 ± 6 cm; 75.5 ± 9.7 kg	G_VBRT1_: 0% VL (14) G_VBRT2_: 10% VL (14) G_VBRT3_: 20% VL (13) G_VBRT4_: 40% VL (14)	2	MPV 70–85% 1-RM	3 sets with 0%, 10%, 20% or 40% VL	8	T-Force	RM	G_VBRT1_: 13.7 ^†^ G_VBRT2_: 18.1 ^†^ G_VBRT3_: 14.9 ^†^ G_VBRT4_: 12.3 ^†^
Shattock et al. [64]	20 (20:0)	22 ± 3 years; 94.3 ± 15.5 kg	G_VBRT_ (10) G_RPE_ (10)	3–4	MPV: 70–85%	3 reps × 8 sets or 4 reps × 6 sets	12	NA	CMJ	G_VBRT_: 8.2 ^†^ * G_RPE_: 3.8 ^†^
									RM_SQ_	G_VBRT_: 7.5 ^†^ * G_RPE_: 3.5 ^†^
									RM_BP_	G_VBRT_: 7.7 ^†^ G_RPE_: 3.8 ^†^
									Sprint 10 m	G_VBRT_: −0.4 G_RPE_: 0.5
									Sprint 20 m	G_VBRT_: −0.4 G_RPE_: 0.1
Rodriguez-Rosell et al. [30]	25 (25:0)	22.6 ± 3 years; 74.5 ± 10 kg	G_VBRT1_: 10% VL (12) G_VBRT2_: 30% VL (13)	2	MPV 0.84–0.60	3 sets with 10% or 30% VL	8	T-Force	1-RM	G_VBRT1_: 17.9 ^†^ G_VBRT2_: 14.9 ^†^
									CMJ	G_VBRT1_: 9.2 ^†^ G_VBRT2_: 5.4 ^†^
									Sprint 10 m	G_VBRT1_: −0.6 ^†^ G_VBRT2_: 0.7 ^†^
									Sprint 20 m	G_VBRT1_: −1.5 ^†^ G_VBRT2_: −0.4 ^†^
Rodriguez-Rosell et al. [29]	33 (33:0)	22 ± 3 years; 72 ± 8 kg	G_VBRT1_: 10% VL (11) G_VBRT2_: 30% VL (11) G_VBRT3_: 45% VL (11)	2	MPV 1.07–0.84	3 sets with 10%, 30% or 45% VL	8	T-Force	RM_SQ_	G_VBRT1_: 22.1 ^†^ G_VBRT2_: 22.0 ^†^ G_VBRT3_: 15.4 ^†^
									CMJ	G_VBRT1_: 12.0 ^†^ * G_VBRT2_: 5.0 ^†^ G_VBRT3_: 4.6 ^†^
									Sprint 10 m	G_VBRT1_: −3.4 † G_VBRT2_: −1.1 G_VBRT3_: 0.0
									Sprint 20 m	G_VBRT1_: −2.3 ^†^ G_VBRT2_: −1.9 ^†^ G_VBRT3_: −0.6
Rodriguez-Rosell et al. [65]	32 (32:0)	23.2 ± 3 years; 75.8 ± 9 kg	G_VBRT1_ LP: (16) G_VBRT2_ UP: (16)	2	MPV 1.16–0.68	3 sets with 15% VL	8	T-Force	1-RM	G_VBRT1_: 14.2 ^†^ G_VBRT2_: 8.5 ^†^
									CMJ	G_VBRT1_: 12.2 ^†^ G_VBRT2_: 8.8 ^†^
Riscart-Lopez et al. [66]	43 (43:0)	22.9 ± 4.8 years; 71.7 ± 7.6 kg	G_VBRT1_ LP: (11) G_VBRT2_ UP: (10) G_VBRT3_ RP: (11) G_VBRT4_ CP: (11)	2	MPV 1.14–0.59	3 sets with 20%VL	8	T-Force	1-RM	G_VBRT1:_ 17.2 ^†^ G_VBRT2_: 10.9 ^†^ G_VBRT3_: 18.0 ^†^ G_VBRT4_: 15.23 ^†^
									CMJ	G_VBRT1_: 5.2 ^†^ G_VBRT2_: 8.0 ^†^ G_VBRT3_: 10.8 ^†^ G_VBRT4_: 7.2 ^†^
									Sprint 20 m	G_VBRT1_: −2.0 ^†^ G_VBRT2_: −1.3 ^†^ G_VBRT3_: −2.0 ^†^ G_VBRT4_: −1.6 ^†^
Jiménez-Reyes et al. [67]	24 (24:0)	23.1 ± 3 years; 73.6 ± 6 kg	G_VBRT1_ AL: (13) G_VBRT2_ NAL: (11)	2	MPV 1.13–0.68 1-RM 50–80%	2–8 reps × 3–4 sets	8	T-Force	1-RM	G_VBRT1_: 12.7 ^†^ * G_VBRT2_: 28.9 ^†^
									CMJ	G_VBRT1_: 7.9 ^†^ * G_VBRT2_: 16.1 ^†^ *
									Sprint 10 m	G_VBRT1_: −1.2 ^†^ G_VBRT2_: −2.2 ^†^
									Sprint 20 m	G_VBRT1_: −0.95 ^†^ * G_VBRT2_: −1.99 ^†^

Data are expressed as means ± SD. The change values are expressed as percentage according to the formula: ((post-pre)/pre) × 100. To facilitate convention, the groups are represented using the capital “G”. AL: adjusted load; BP: full bench press; BP2/3: two-thirds bench press; BP1/3: one-third bench press; CMJ: Countermovement Jump; CP: constant programming; DL: dead lift; F: females; HV: high velocity; LP: linear programming; M: males; MPV: mean propulsive velocity (m∙s^−1^); MV: medium velocity; PBT: percentage-based training; RPE: rating of perceived exertion; FSQ: full squat; NA: not available; NAL: non-adjusted load; OP: overhead press; PP: Wingate peak power; 1-RM: one-repetition maximum; RP: reverse programming; SQ: full back squat; UP: undulating programming; VBRT: velocity-based resistance training; VL: velocity loss. ^†^ Significant difference in comparison to baseline; * significant difference in comparison to the other group.

## Data Availability

The data supporting this systematic review are from previously reported studies and datasets, which have been cited.
